# Endothelial cells influence the osteogenic potential of bone marrow stromal cells

**DOI:** 10.1186/1475-925X-8-34

**Published:** 2009-11-17

**Authors:** Ying Xue, Zhe Xing, Sølve Hellem, Kristina Arvidson, Kamal Mustafa

**Affiliations:** 1 Department of Clinical Dentistry-Center for Clinical Dental Research, University of Bergen, Bergen, Norway; 2 Centre for International Health, Faculty of Medicine and Dentistry, University of Bergen, Bergen, Norway; 3 Department of Clinical Dentistry-Section for Oral and Maxillofacial Surgery, University of Bergen, Bergen, Norway

## Abstract

**Background:**

Improved understanding of the interactions between bone cells and endothelial cells involved in osteogenesis should aid the development of new strategies for bone tissue engineering. The aim of the present study was to determine whether direct communication between bone marrow stromal cells (MSC) and human umbilical vein endothelial cells (EC) could influence the osteogenic potential of MSC in osteogenic factor-free medium.

**Methods:**

After adding EC to MSC in a direct-contact system, cell viability and morphology were investigated with the WST assay and immnostaining. The effects on osteogenic differentiation of adding EC to MSC was systematically tested by the using Superarray assay and results were confirmed with real-time PCR.

**Results:**

Five days after the addition of EC to MSC in a ratio of 1:5 (EC/MSC) significant increases in cell proliferation and cellular bridges between the two cell types were detected, as well as increased mRNA expression of alkaline phosphatase (ALP). This effect was greater than that seen with addition of osteogenic factors such as dexamethasone, ascorbic acid and β-glycerophosphate to the culture medium. The expression of transcription factor Runx2 was enhanced in MSC incubated with osteogenic stimulatory medium, but was not influenced by induction with EC. The expression of Collagen type I was not influenced by EC but the cells grown in the osteogenic factor-free medium exhibited higher expression than those cultured with osteogenic stimulatory medium.

**Conclusion:**

These results show that co-culturing of EC and MSC for 5 days influences osteogenic differentiation of MSC, an effect that might be independent of Runx2, and enhances the production of ALP by MSC.

## Introduction

The goal of bone tissue engineering is the generation of new bone from osteogenic cells, supported with biocompatible, biodegradable three-dimensional scaffolds [1,2]. The type of osteogenic cell most appropriate for bone tissue engineering has yet to be determined. Bone marrow stromal cells (MSC) were first isolated by Friedenstein & Owen [3,4] and are regarded as the main source of bone progenitor cells in skeletal tissues. As MSC can be isolated from adult bone marrow and expanded *in vitro *without loss of differentiation potential, they are suitable candidates for bone tissue engineering [1,5-7].

Bone is a highly vascularised tissue and close communication between blood vessels and bone cells is essential for maintaining skeletal health [8,9]. Osteogenesis is a complicated process influenced by physiological conditions, cell-to-cell interactions, extracellular matrix formation and surrounding vascularization. After a tissue-engineered bone construct is implanted *in vivo*, induction of initial vascularization is important [2]: in particular, the survival of osteogenic cells at the center of scaffolds is often threatened by the limited extent of initial vascularization [10]. Thus in bone tissue engineering, the addition of vascular cells might offer several advantages over the use of osteogenic cells alone [11].

Studies of the interaction between osteoblasts and endothelial cells (EC) have demonstrated the formation of microvessel-like structures and gap junction communications [12,13]. Paracrine factors such as vascular endothelial growth factor (VEGF) are implicated as mediators in these interactions [11,14]. EC derived from microvasculature, umbilical veins or large blood vessels have been used for generation of capillary-like structures and vessels *in vitro *[15,16].

The relationship between bone cells and EC in osteogenesis has yet to be fully elucidated. Therefore, the aim of the present study was to determine whether direct communication between the two cell types in an osteogenic factor-free medium could influence osteogenic differentiation of MSC.

## Materials and methods

### Cell culture and maintenance

Primary human MSC (StemCell Technologies, Vancouver, BC, Canada) were cultured in MesenCult^® ^complete medium (StemCell Technologies) following the manufacturer's instructions. Purity of the cells was confirmed by flow cytometry, which showed that > 90% of the cells expressed CD29, CD44, CD105, CD166 and < 1% expressed CD14, CD34 and CD45. The cells were expanded in culture for use in the experiments with an osteogenic stimulatory medium (StemCell Technologies) that was freshly made and supplemented with dexamethasone, ascorbic acid and β-glycerophosphate according to the manufacturer's instructions.

Primary human umbilical vein endothelial cells were obtained from Lonza (Clonetics^®^, Walkersville, MD). In accordance with the manufacturer's instructions, the cells were expanded in EGM Medium (Clonetics^® ^EGM^®^) containing 500 ml of Endothelial Cell Basal Medium and the following growth supplements: BBE, 2 ml; hEGF, 0.5 ml; Hydrocortisone, 0.5 ml; FBS, 10 ml; GA-1000, 0.5 ml.

### Direct contact co-culture of EC and MSC

EC and MSC were trypsinized separately and then co-cultured in 6-well plates (Nunclon, Roskilde, Denmark) and plated at a ratio of 1:5. The cell density was 2 × 10^3^/cm^2 ^EC and 1 × 10^4^/cm^2 ^MSC in a mixed, osteogenic factor-free medium. MSC were also grown alone in both osteogenic and osteogenic factor-free medium. The culture medium was changed after 3 days.

### Immunostaining

After 1, 3 and 5 days of culture, cells grown on Ø 18 mm coverslips (VWR international, West Chester, PA) were rinsed in phosphate buffered saline (PBS) and fixed with 4% paraformaldehyde for 10 min at room temperature. In order to distinguish the two cell types, MSC were labelled with FITC-CD90 (BD Pharmingen™, San Jose, CA) and EC with TRITC-UEA I lectin (Vector Laboratories, Burlingame, CA). In brief, TRITC-UEA I lectin (1:500) was first added and incubated for 1 h at room temperature. After washing with PBS, CD 90 antibody was added and incubated for 1 h at room temperature. The nuclei were stained with DAPI (Molecular Probe™, UK) solution (1 μg/ml) and washed with PBS. The samples were mounted and examined by fluorescence microscopy (Nikon 80i, Tokyo, Japan).

### SEM

The cells were grown on coverslips for 1 and 5 days, and then prepared for scanning electronic microscopy. The samples were rinsed in 2.5% glutaraldehyde in α-MEM without serum and fixed for 30 min at room temperature. The samples were then fixed in 2.5% glutaraldehyde in 0.1 M Na-cacodylate PH 7.2 with 0.1 M sucrose for further 30 min at room temperature. The samples were treated with 1% osmium tetroxide in distilled water for 1 h, followed by dehydration through a graded series of ethanol from 70%, 80%, 95% and 100%. Critical point drying was carried out using CO_2 _as transitional fluid. The samples were mounted on aluminum holders and coated with a 10 nm conducting layer of gold platinum. The samples were examined in the scanning electronic microscope (Jeol JSM 7400F, Tokyo, Japan) using a voltage of 10 kV.

### Cell viability and proliferation test

Cell proliferation and cell viability was analyzed using a colorimetric assay for quantification of cleavage of the tetrazolium salt WST-1 (Roche Molecular Biochemicals, Hannheim, Germany) by mitochondrial dehydrogenases in viable cells. The dye formed can be quantified by spectrophotometer and is directly correlated to the number of metabolically active cells in the culture. The cells were grown in 96-well plates (Nunclon, Roskilde, Denmark) for 1, 3 and 5 days. After each incubation period, the cells were incubated for a further 12 h at 37°C with 100 μl medium containing 10 μl WST-1 reagent. The samples were shaken for 1 min and absorbance at 450 nm was measured by a microplate spectrophotometer (BMG LABTECH, GmbH, Germany). Fresh medium was used as a negative control.

### Total RNA extraction

Total RNA was isolated from 5-day-old cultures using Trizol^® ^reagent (Gibco BRL, Carlsbad, CA) combined with E.Z.N.A.™ Tissue RNA isolation kit (Omega Bio-Tek, Norcross, GA) according to the manufacturer's protocol. Total RNA was quantified using a Nanodrop Spectrophotometer (ThermoScientific NanoDrop Technologies, Wilmington, DE). The quality of the RNA preparation was verified by 1% agarose gel electrophoresis.

### RT^2 ^Profiler PCR Array of osteogenesis

Contamination of genomic DNA was removed from total RNA samples by DNase I digestion prior to first-strand synthesis. cDNA synthesis was performed with the RT^2 ^PCR array First Strand Kit (SuperArray Bioscience Corporation, Frederick, MD). Human Osteogenesis RT^2 ^Profiler PCR Array and RT^2 ^Real-time SyBR Green/ROX PCR Mix were purchased from SuperArray Bioscience Corporation. PCR was performed on ABI StepOne system (Applied Biosystems). The ΔΔCt method was used for data analysis, and each gene fold-changes was calculated as difference in gene expression between co-cultured MSC and MSC cultured alone.

### Real time Polymerase chain reaction (PCR)

The reverse transcription reaction was performed according to the manufacturer's instructions using the High Capacity cDNA Archive Kit (Applied Biosystems). From 300 ng to 1 μg total RNA was obtained and mixed with reverse transcriptase (RT) buffer, random primers and Multiscribe RT. Mixtures of fresh RNA samples were serially diluted and used as standards to build up the standard curve.

Real time qPCR was used to detect mRNA levels of glyceraldehyde-3-phosphate dehydrogenase (GAPDH), alkaline phosphatase (ALP), Collagen Type I (Col I) and Runt-related transcription factor 2 (Runx2).

Quantitative RT-PCR (qRT-PCR) was performed under standard enzyme and cycling conditions on a StepOne system using four TaqMan gene expression assays: Hs01029142_m1 (ALP), Hs00164099_m1 (Col I), Hs00231692_m1 (Runx2), and TaqMan Pre-Developed Assay GAPDH (4333764T). cDNA corresponding to 6 ng of mRNA was used in each PCR reaction. Amplification was carried out in 96-well thermal cycle plates on a StepOne system (Applied Biosystems). The Data were analyzed by StepOne software using the Relative Standard Curve method. GAPDH was used as an endogenous control.

### Statistical analysis

All the experiments were repeated three times using 3 donors to provide MSC and a pooled human EC. The data were expressed as mean ± SD for n = 3. The data were tested for normal distribution and variance homogeneity, using one-way ANOVA. Differences between means were considered statistically significant when p < 0.05. For statistical analysis, SPSS 15.0 software was used.

## Results

### Immunostaining

**Figure 1:**
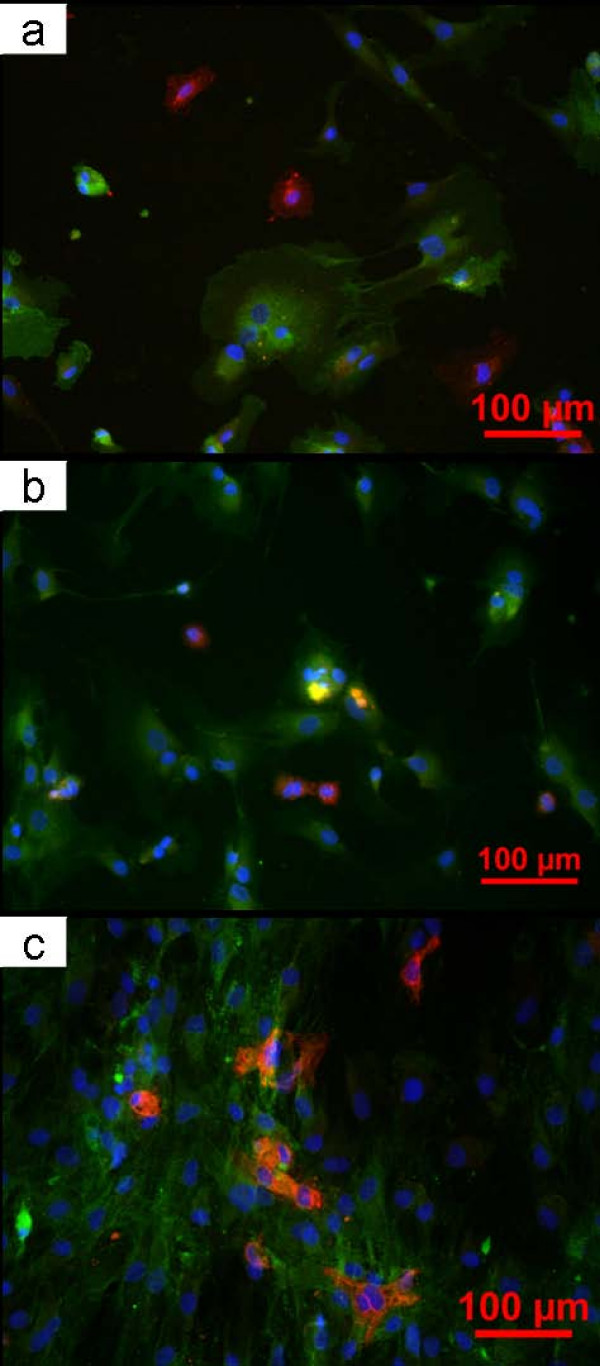
**Cells grown on the coverslips for 1 (a), 3 (b) and 5 (c) days: MSC were labeled with CD90 (green), EC with UEA I (red) and nuclei with DAPI (blue)**.

**Figure 2:**
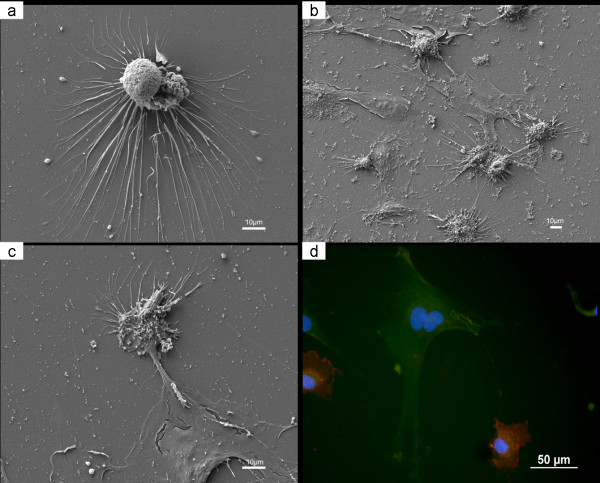
**Morphology of cell communications between co-cultured MSC and EC: SEM showing morphology of EC after 5 days (a); SEM showing MSC and EC after 5 days (b); cellular bridge between MSC and EC disclosed by SEM (c) and by immunostaining (d)**.

MSC were labelled with CD90 (green) and EC with UEA 1. All the nuclei were labelled with DAPI (Blue). MSC were osteoblast-like in shape and in general the EC had retained their cobblestone-like morphology. During 5 days' incubation, there was no obvious change in shape of MSC. It could be observed that the number of MSC gradually increased within 5 days (Fig. 1). Higher magnification disclosed cellular bridges between the two cell types after 1 day (Fig. 2d).

### SEM

After 5 days, MSC showed larger spatial spreading and extracellular matrix formation and EC were surrounded by more cell foci (Figs. 2a and 2b). Under co-culture conditions, the cells were attached and spread on the culture plate (Fig. 2b). The phenomenon of cellular bridges between MSC and EC disclosed by immunofluorescent staining was also detected by SEM imaging after 1 day (Figs. 2c and 2d).

### Cell proliferation

**Figure 3:**
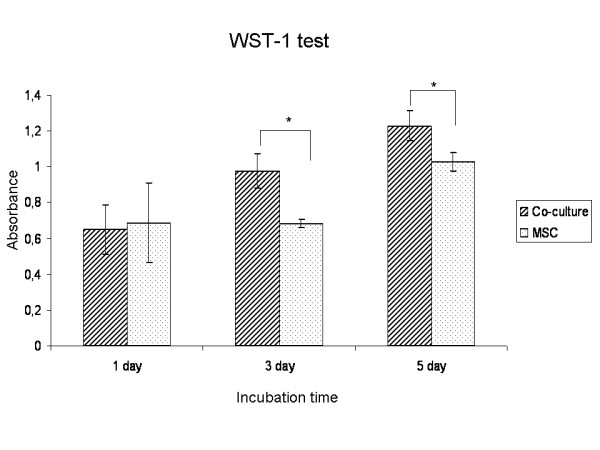
**Results obtained by WST-1 assay to describe cell viability and proliferation after 1, 3 and 5 days**. The data were representative for all donors and presented as mean ± standard deviations. * P < 0.05 (compared with MSC group).

In general, the results indicated that cell numbers increased during the incubation period (Fig. 3). There was no statistical difference between the two groups after day 1. After 3 and 5 days of incubation, the mitochondrial activity of cells under co-culture conditions was significantly higher compared to the control group.

### RT^2 ^Profiler PCR Array of osteogenesis

**Figure 4:**
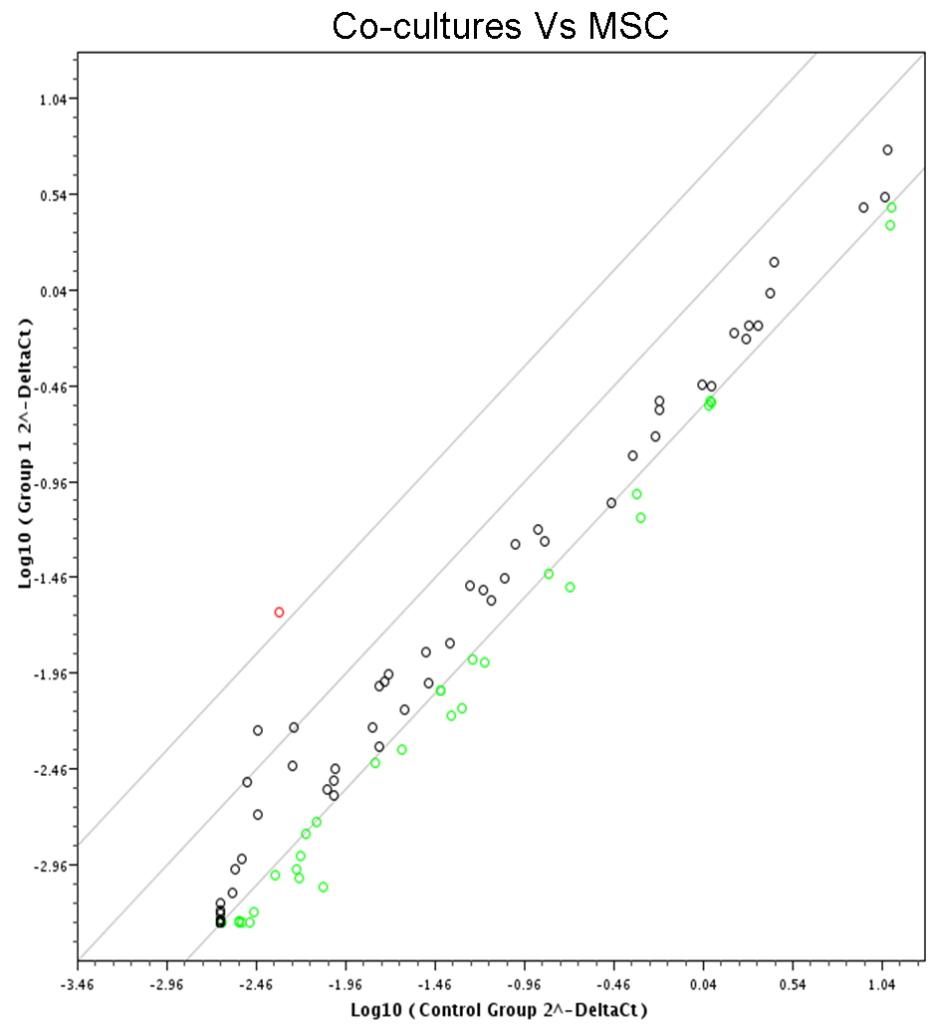
**RT^2 ^Profiler PCR Array of osteogenesis after 5 days**. Overview of scatter plot on expression of 84 osteogenesis genes: red dot is up-regulated gene and green dot is down-regulated genes.

**Figure 5:**
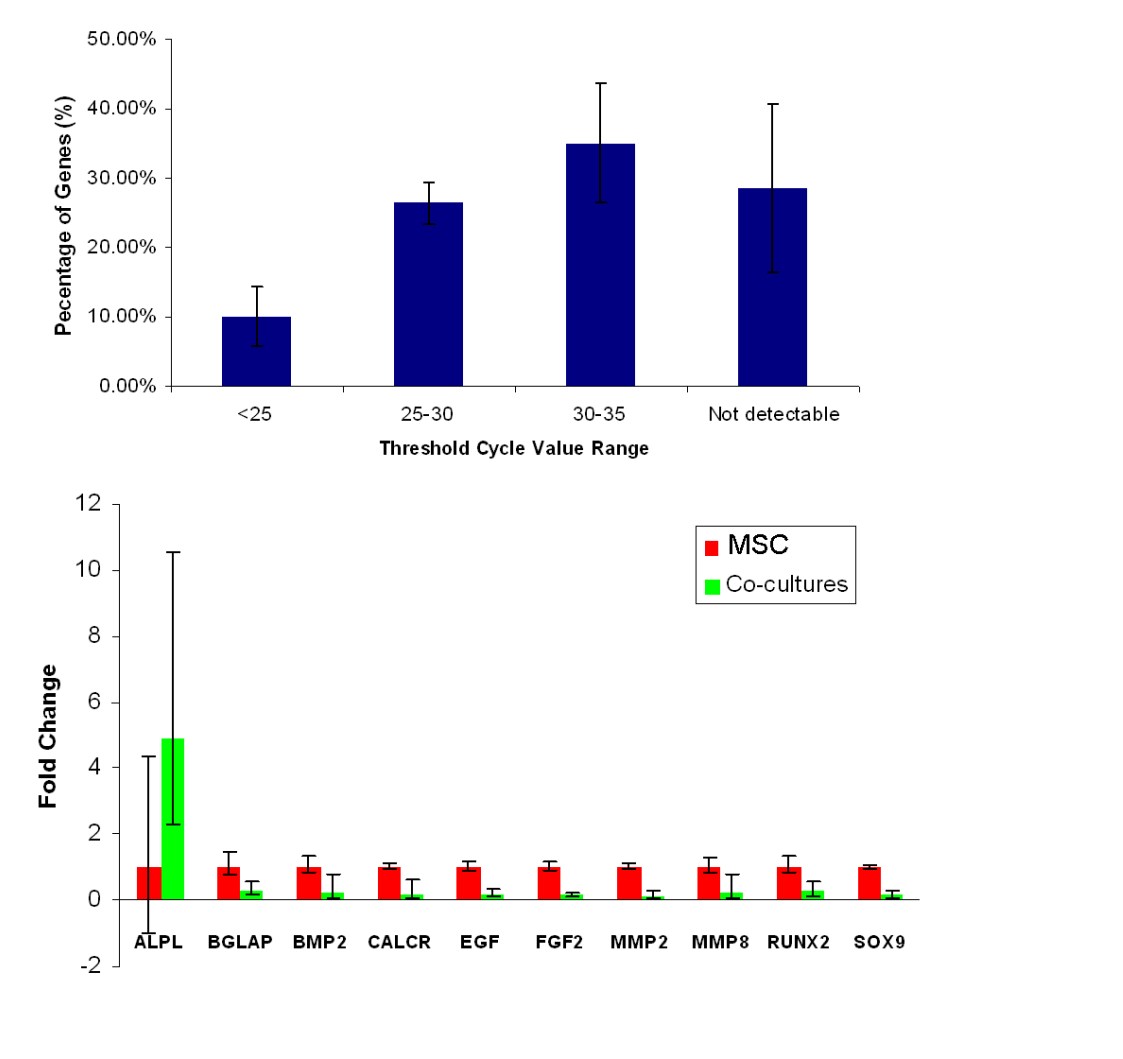
**RT^2 ^Profiler PCR Array of osteogenesis after 5 days**. (a): Histogram showing the mean cycle threshold distribution for MSC group at 5 days. Low CT values (< 25) represent genes at high transcript copy number. CT values of greater than 35 were considered to be outside the detection threshold of the system. (b): Fold changes of genes by co-culturing group compared with MSC group. MSC group was used as reference group and set as 1. Results were reported as fold changes ± fold-difference (2^-ΔΔCt ^± 2^-(ΔΔCt ± SD)^). * P < 0.05.

Samples were screened for the expression of 84 genes linked to osteoblast differentiation and bone metabolism. The data reflected that MSC had a pattern of constitutive expression for the majority of osteogenic genes (Fig. 4 and 5a). In total, 25 genes of MSC showed either up- or down- regulated expression after treatment with endothelial cells for 5 days (Fig. 4). Of these, 5 genes were statistically significantly different (Fig. 5b). After co-culturing with EC for 5 days, MSC had a 4.92-fold up-regulation of ALP. The expressions of RunX2 and Col I were down-regulated, but the differences were not significant (P > 0.05). At this time point, the detected level of bone gamma-carboxyglutamate protein (BGLAP) was lower (Ct > 33). The following genes related to chondrocyte differentiation were down-regulted: SRY-box containing gene 9 (Sox 9), 6.43-fold; Matrix metallopeptidase 2 (MMP_2_), 6.9-fold; Epidermal growth factor (EGF), 5.63-fold; Fibroblast growth factors 2 (FGF_2_), 6.33-fold.

### PCR results

**Figure 6:**
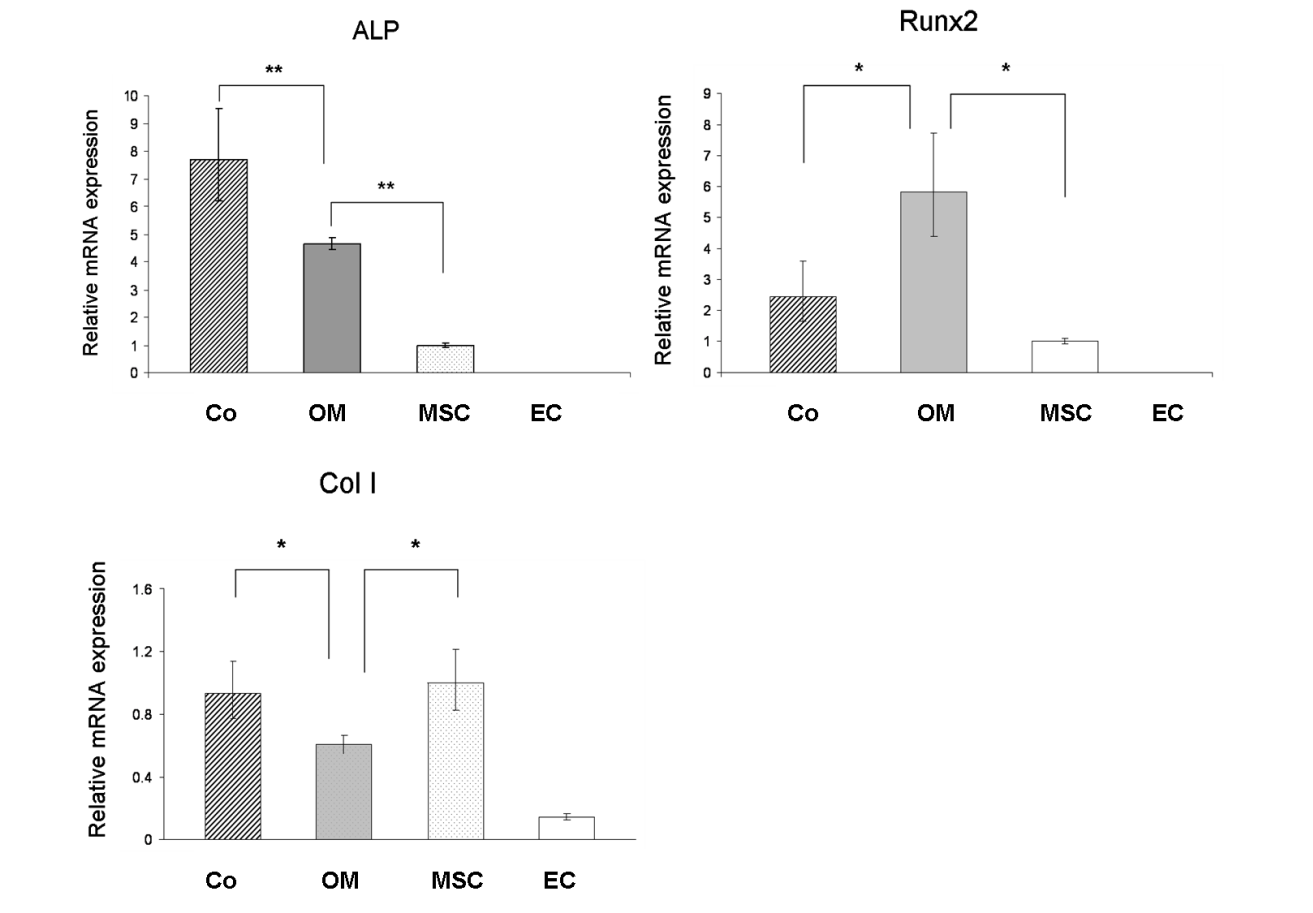
**mRNA Expression after 5 days by real time PCR**. There were four groups: Group 1: MSC co-cultured with EC (Co); Group 2: MSC induced by osteogenic stimulatory medium (OM); Group 3: MSC in mono-culture without osteogenic stimulatory supplements (MSC) and Group 4: EC only (EC). Results were reported as fold changes ± fold-difference (2^-ΔΔCt ^± 2^-(ΔΔCt ± SD)^). The y-axis showed mRNA level in logarithmic scale for ALP, RunX2 and Col I. A significant upregulation of ALP expression was found in MSC/EC co-cultures compared with MSC alone. All the data were normalized with endogenous control GAPDH. ** P < 0.01 * P < 0.05.

In order to confirm the Superarray results, real-time PCR was performed independently and 5 day osteogenic stimulatory medium (OM)-induced MSC were used as a positive control (Fig. 6).

ALP and Col I expression were used as early markers of osteogenic differentiation. After 5 days, there was a statistically significant (P < 0.01) increase of ALP mRNA levels in MSC/EC co-cultures compared with MSC alone. The addition of osteogenic stimulatory medium to MSC also resulted in increased ALP expression, but interestingly this increase was significantly less than the expression of ALP by MSC co-cultured with EC. There was almost no detectable expression of ALP in monocultures of EC.

Adding osteogenic stimulatory medium significantly decreased Col I expression compared with MSC with or without EC (P < 0.05). The addition of EC to MSC caused no significant difference in expression of Col I.

The mRNA expression of Runx2 increased significantly after addition of osteogenic stimulatory medium compared with expression by MSC alone or with EC (P < 0.05). Addition of EC to MSC caused no significant difference in expression of Runx2.

## Discussion

The present *in vitro *study tested the hypothesis that direct communication with endothelial cells might enhance the osteogenic potential of MSC. Under osteogenic inductive medium culture conditions, MSC progress from immature progenitor cells through the osteoblastic lineage to become osteocytes [17,18]. Medium supplements such as dexamethasone (DEX), ascorbic acid and β-glycerolphosphate have been shown to influence the development of MSC into more highly differentiated phenotypes. However, the underlying mechanism has not been clarified. Differentiation of stem cells towards osteogenesis requires interaction with other cells at a crucial moment [3]. In most of the co-culture studies to date, cells have been treated with osteogenic stimulatory medium [14,19,20], whereas the present study was undertaken without the addition of osteogenic supplements to MSC or to the co-culture group.

In the co-culture studies cited above [14,19,20], either conditioned medium was used or the two cell types were separated by a permeable membrane inserted into the culture plate. The use of inserts in the non-contact culture systems facilitates analysis and eliminates the risk of contamination between the two cell types. Soluble factors secreted by one cell type can move through the inserts to induce cell differentiation. The major disadvantage of using membranes or conditioned medium is that they prevent intimate physical contact or regulatory communication between the cell types. This limitation of signal transduction is in contrast to conditions *in vivo *[11]. For these reasons, the direct contact culture system was adopted in the present study. To reduce the influence of a mixture of cells on gene analysis, the present studies were designed using a 1:5 (EC:MSC) cell mix for 5 days of culture. Different EC:MSC ratios were co-cultured in pilot studies (data was not showed) and it was found that EC could grow well and exist as a continus stimulatory factor for MSC in the 1:5 ratio which was closely mimics the *in vivo *situation [11].

The SEM and immunostaining results indicated that there might be communication between the two cell types after 5 days of culture. The design of the present study did not include specific tracing of such communication and the specific signal transduction pathways between two cell types could therefore not be defined. In a previous study, microscopic examination disclosed the presence of tunneling nanotubes (TNT), which might participate in cell-to-cell communication between the two cell types [21]. The communication suggested by the present study might be attributable to the formation of TNT structures, but further investigation is required for confirmation.

The WST-1 assay proliferation results which allow quantification of mitochondrial metabolism showed that addition of EC did not inhibit cellular growth of MSC after 3 and 5 days of co-culture. Similar results have also been reported from previous studies using different co-culture methods and ratios [14,19,20]. Bone modelling is initiated by osteoblastic differentiation of mesenchymal cells into preosteoblasts which then differentiate into functional osteoblasts producing bone matrix proteins [22]. Collagen type I is one of the major extracellular matrix proteins in fibrous tissue and bone [23]. In the present study, the PCR results showed no significant change in Col I expression after addition of EC to MSC.

ALP is commonly expressed in bone, liver, kidney, brain, lung and neutrophils [24]. Although ALP isolated from bone tissue is considered to have a major role in skeletal mineralization, the specific biologic functions of the bone isoforms are currently unknown. Importantly, in the early stages of osteoblast-mediated mineralization, bone ALP is considered to have an important function in the removal of inorganic pyrophosphate, a potent inhibitor of mineralization [24]. ALP is often used as a biochemical and histochemical marker for identification and evaluation of osteogenesis. Interestingly in the present study, there was nearly a 5-fold increase in ALP expression when MSC were co-cultured with EC of less than 20% of the total cell population. This increase was demonstrated by culturing the cells in osteogenic factor-free medium using both Superarray and PCR methods. Furthermore, the PCR results showed that after 5 days of culturing MSC and EC, the effect induced by EC was greater than that the effect induced by osteogenic stimulatory medium.

During osteogenesis from mesenchymal progenitors, various extrinsic factors such as dexamethasone and cytokines regulate osteoblastic differentiation. Two major pathways for osteogenesis are recognized; one is Runx2 dependent and the other is Runx2 independent [25]. To investigate the possible reasons underlying the changes in ALP expression described above, the key transcription factor Runx2 was tested by PCR. Within 5 days of induction, the addition of osteogenic stimulatory medium to MSC significantly induced the expression of Runx2. However, co-culturing of MSC with EC did not influence Runx2 expression. Runx2/cbfa1 was used as an early-stage transcription factor for osteoblast differentiation. In Runx2/cbfa1 null mice, osteoblast differentiation is arrested in both the endochondral and intramembranous skeleton [26,27]. It has been shown that Runx2 plays a role in the commitment-step to osteo-chondro progenitor cells [28]. A previous *in vitro *study has shown that dexamethasone and Runx2 could synergistically induce osteoblastic differentiation [29]. The main component of the osteogenic stimulatory medium was dexamethasone and the results demonstrated that Runx2 played a crucial role during dexamethasone-induced osteoblastic differentiation.

The findings of the present study indicate that up-regulation ALP from MSC was induced by EC and might be independent of Runx2 but the identification of other potential pathways will require further investigation. However, with the low level of bone gamma-carboxyglutamate protein (BGLAP) expressed by MSC, it is unlikely that these immature cells had the potential to undergo terminal differentiation within 5 days of co-culture with EC. Therefore, our results suggest that in 5 days' induction, EC could direct MSC to the early stage of osteogenic cells. Since mesenchymal stem cell could differentiate into chondrocytes through along a multistep, recruitment of chondrogenitor cells are related with the communication of neighboring cells as EC [30]. It is known that SRY-box containing gene 9 (Sox 9) is a transcription factor that is completely required for chondrocyte differentiation associated with fibroblast growth factors (FGFs) [30,31]. MMP2 has wide substrate specificity for cartilage matrix constituents [32]. The Superarray disclosed that the genes related to chondrocyte differentiation (Sox 9, MMP_2_, FGF_2_) were down-regulated, suggesting that EC might provide important signals for chondrocytic differentiation and have an important influence on chondrocyte commitment and maturation.

Further investigation is warranted to determine the effect on the differentiation potential of MSC of varying the ratio of EC to MSC and also to clarify the role of EC in the regulation of MSC differentiation into osteoblasts during long-term incubation.

## Conclusion

Under osteogenic factor-free conditions, the addition of EC induced the expression of ALP by MSC in a short incubation time (5 days). The ALP expression was even higher than that resulting from the addition of osteogenic stimulatory medium to MSC. Based on these preliminary results it is concluded that co-culturing MSC with EC influenced osteogenic differentiation of MSC and this effect might be independent of Runx2. Future studies will be directed towards the induction of ALP and the regulatory role of EC in the differentiation of MSC into osteoblasts.

## Competing interests

The authors declare that they have no competing interests.

## Authors' contributions

YX carried out the cell cultures, performed the genetic analysis and drafted the manuscript. ZX carried out the immunoassays and performed the statistical analysis. SH and KA supervised the project, and participated in revisions of the manuscript. KM, supervisor, participated in its design, and contributed to the discussion and interpretation of the results and helped to draft the manuscript. All authors read and approved the final manuscript.
